# Geospatial Visualisation of Distance to General Practitioner Facilities with Population Density Patterns in the United Kingdom

**DOI:** 10.3390/epidemiologia7030085

**Published:** 2026-06-17

**Authors:** Mathieu Di Miceli

**Affiliations:** School of Biomedical Sciences, Faculty of Biological Sciences, University of Leeds, Leeds LS2 9JT, UK; m.c.dimiceli@leeds.ac.uk

**Keywords:** health services indicators, measurements, distance to primary care, United Kingdom

## Abstract

**Background/Objectives**: To quantify geographical distances to nearest general practitioner (GP) services for all household postcodes in the United Kingdom. **Methods**: We mapped household postcodes in the United Kingdom and computed distances to nearest GP practice, using centroid geographical coordinates (latitude and longitude). We also analysed the total number of GP practices throughout local area districts (LADs) in relation to population density. **Results**: As of December 2023, there were 7965 active GP practices across the UK, serving a total registered population of over 73 million patients. Analysis of 1.78 million household postcodes revealed that 98.8% were within 10 km of a GP practice (measured as a straight-line). The most distant postcode was in the Shetland. Throughout the UK, population density was weakly or strongly correlated with number of GP practices in the different LADs, with wide variations, and the strongest correlation observed in Northern Ireland. **Conclusions**: In the UK, geographical proximity to nearest GP practice was found to be within 10 km for the vast majority of residents. Weak to strong correlations between population density and number of GP practices were observed. Future work should quantify the impact of both staffing capacity and public transport availability on distance to GP surgeries across the UK, to better characterise structural determinants of primary care accessibility.

## 1. Introduction

Primary care serves as the foundation of modern healthcare systems, providing first-contact, continuous and coordinated care for a wide range of health needs. Timely access to primary care services is associated with improved health outcomes, reduced healthcare inequalities, and lower rates of avoidable hospital admissions. In the United Kingdom, general practitioner (GP) practices play a central role in delivering primary care, acting as gatekeepers to specialist services while managing the majority of patient consultations within the National Health Service (NHS). Estimated data suggest that there were almost 377 million appointments made in 2025 [1], highlighting how crucial primary care is for initial non-urgent patient triage. As demand for healthcare continues to increase due to population growth, ageing demographics and the rising prevalence of chronic disease, ensuring equitable access to GP services has become an important policy and public health priority. Healthcare accessibility is a multidimensional concept encompassing factors such as workforce [2], service availability [3], affordability [4] and geographical accessibility [5]. Among these, geographical accessibility remains a key determinant of healthcare utilisation, as greater travel distances and longer journey times can act as barriers to seeking care. These challenges may be particularly pronounced in rural and remote communities, where healthcare facilities are often more sparsely distributed.

Accessing appointments at GP practices can be challenging due to the patient-to-staff ratio, which rose from 2120 patients per fully qualified GP in 2018 to 2260 in 2022 (in England) [6]. However, this ratio decreases when all GPs are considered, from 1800 in 2018 to 1720 in 2022 [6]. Since universal health coverage is included in the United Nations Sustainable Goals (“ensure healthy lives and promote well-being for all at all ages”, target 3.8 for universal coverage), access to GP practices is key to ensure prevention and treatment of diseases. In 2017, the National Health Service (NHS) in England issued a document explaining the rollout of an improved access scheme [7]. When accessing GP appointments, most patients (72%) preferred online booking systems [8]. Telephone and video consultations are possible, with the latter only representing a minority [8]. Face-to-face appointments were highly correlated to patient satisfaction, with half of patients (50.6%) securing appointments on the same day or on the following day [9]. The latest figures in England report that 44.2% of appointments were made on the same day, 63.8% were face-to-face, 44.1% were seeing a GP (19.5% for nurses) and 89.9% of all appointments were attended by patients [10]. In 2025, the number of GP practices in England was 6248 (reduced from 6424 in 2023, including 6398 active) for a total of 63.77 million patients registered [10]. In 2025, 29.2–32.9 million appointments were made in a month, with over 362 thousand home visits [10]. In April 2025, the monthly volume of appointments for GP consultations was just over 13 million [10]. Acute consultations represent 18.5% of appointments, 26.1% are devoted to routine assessments, while walk-ins remain very rare (0.1%) [10]. Interestingly, clinical triage represents 11.2% of all appointments.

The latest figures on the GP workforce consisted of 38,018 full-time equivalents (FTEs), which represent a 2.1% rise from the year before [11]. Fully trained doctors represent 28,249 FTEs, of which 27,676 are permanent staff [11]. In England, nurses represent 16,786 FTEs, with a 0.9% decrease from the year before, and administrative staff represent 76,022 FTEs [11]. The number of physician associates (807 FTEs), pharmacists (1835 FTEs) and healthcare assistants (6910 FTEs) are relatively stable when compared to the previous year [11]. It is also worth noting that the majority of GPs (74%) were trained in the UK, a minority trained in the European Union (4%) and a significant proportion (19%) trained elsewhere [11].

In 2016, NHS England launched a monetary incentive for GPs to settle in communities deprived of general practitioners, linked to rural areas [12]. From 2004 to 2013, access to GP practices for the most deprived patients increased from 54.0 to 60.5 FTEs [13]. Furthermore, distance and deprivation correlate with volume of appointments requested by patients [14,15]. In Northern Ireland, 19% of the local areas have patients living within 1.5 km of a GP practice [16]. Another study identified distance as a significant factor for assessing GP practices, but not hospitals, with not owning a car presented as a significant factor [17]. More recent data indicate that 81.2% of the population lives within walking distance of the GP practice [18].

Out of 737 thousand cross-sectional cancer records examined, 88.2% of patients had access to a GP practice in a less than a 10 min drive, whilst 0.6% needed to drive for more than 30 min [19]. In the same study, distance to the nearest GP practice was associated with poorer breast, colorectal, lung, prostate, stomach and ovarian cancer survival (higher risk of death) [19], which raises concerns as to whether a correlation could exist between distance to the nearest GP practice and prognostic to specific diseases, especially in remote rural areas. This is further supported by a study in the United States showing that cancer screening is less frequent in rural settings (compared to urban) [20]. Other studies have been published on the links between geographical distance and access to health services. These include access to accident and emergency departments in Wales [21], prenatal clinics in New York [22], community hospitals in Michigan [23], mental health facilities in the United States [24] and interventions for acute myocardial infarctions in Alberta [25]. Finally, another study assessed travel time to the nearest healthcare facility for the entire world, with some areas requiring patients to travel for several hours, such as in central Australia, northern Russia, Saharan Africa, Alaska, Greenland, mountainous or forested South America, northern Canada and the mountainous regions of Asia [26].

Equitable access to primary healthcare is a fundamental component of an effective healthcare system, yet geographical barriers can limit patients’ ability to access primary facilities, particularly in rural and remote areas. Despite ongoing concerns regarding pressures on UK primary care provision, there has been limited nationwide evidence quantifying the spatial accessibility of GP practices across all residential locations. Previous studies have often focused on specific regions or populations, leaving a gap in understanding the national distribution of primary services and how these relate to population density. By systematically mapping distances from every household postcode in the UK to the nearest GP practice and examining the distribution of practices across local area districts, this study provides a comprehensive assessment of geographical distance to primary care. Such evidence is essential for identifying potential areas of underserved provision, informing healthcare workforce and infrastructure planning, as well as supporting policies aimed at reducing inequalities in access to primary healthcare services.

## 2. Materials and Methods

### 2.1. Datasets

Open-access data were retrieved from the following providers: the Office for National Statistics (OfNS, https://www.ons.gov.uk accessed on 4 June 2025), NHS Digital (NHSD, https://digital.nhs.uk accessed on 4 June 2025), Public Health Scotland (https://publichealthscotland.scot accessed on 4 June 2025), Health and Social Care Scotland (https://www.hscni.net accessed on 4 June 2025), DataMapWales (https://datamap.gov.wales accessed on 4 June 2025), the Welsh Government (https://www.gov.wales accessed on 4 June 2025) and the Open Geography Portal (OGP, https://geoportal.statistics.gov.uk date of access 4 June 2025.). The list of retrieved datasets is presented in Table 1. The following elements were retrieved: (i) latitude and longitude coordinates of GP practices, (ii) number of GP practices in each local area district (LAD), (iii) density of inhabitants in each LAD, (iv) latitude and longitude coordinates of inhabited postcodes.

### 2.2. Geographical Areas

Data related to the entirety of the United Kingdom were retrieved, which included England, Wales, Scotland and Northern Ireland. However, due to the heterogeneity detected in datasets, measurements, mode of encoding and missing data, some data were analysed at more local levels, such as LAD. To create maps, LAD codes were extracted from the OfNS and paralleled with the LAD19CD (December 2019) *shapefile*. All postcodes (*n* = 1,800,248) were retrieved and transformed to centroid geographical coordinates (latitude; longitude) with Mercator adjustments to account for distances from the equator [27,28,29]. Non-occupied or non-geographic postcodes were excluded (*n* = 18,268), leaving 1,781,980 valid postcodes for subsequent analysis. The Coordinate Reference System (CRS) used in the present study is 4326 (formally EPSG:4326), corresponding to the World Geodetic System of 1984 (WGS 84).

### 2.3. Data Analyses

For all valid UK postcodes, geographical centroid coordinates (latitude and longitude) were retrieved from publicly available repositories (Table 1). Similarly, all UK GP practice postcodes (Table 1) were retrieved with a geographical centroid coordinate (latitude and longitude). Subsequently, the nearest GP practice was identified for each household postcode, using the minimum distance possible. The distances between household postcodes and nearest GP practices were then computed by calculating the difference between the positions of the centroids. Briefly, the first step determines the nearest GP practice for each postcode centroid (*st_nearest_feature* function) by checking a matrix of distances from all available features (documentation). Then, the distance between each postcode is computed (*st_distance* function) by calculating the projected Euclidean distance between the nearest feature and the postcode centroid (documentation). To allow mapping, output measurements were then converted from degree angles (from the *st_distance* function using latitude and longitude coordinates) to kilometres, using the circle arc length formula (with Earth’s radius set to 6371 km). Population density data were extracted (Table 1) and compared to the number of GP practices for each LAD. Data analyses were performed on a personal Asus Notebook laptop (i5-6300HQ, 2.3 GHz, 24 GB of RAM, Windows 10.0.19045 x64).

### 2.4. Data Display

Data displayed on box-and-whiskers represent the 25th–75th percentiles with median values also indicated by a line. Trimmed violin plots are displayed to allow visual representation of data distribution. RStudio [30,31,32] version 4.0.3 was used to analyse and display data (download available at Posit [33]). The following R packages were used: *maps*, *OpenStreetMap*, *sf*, *scales*, *ggmap*, *ggplot2*, *ggpubr*, *tidyverse*, *rstatix*, *viridis*, *rgeos*, *rgdal*, *maptools*, *terra*, *postcodesioR*, *readxl*, *xlsx*, *dplyr* and *patchwork*, all available at the Comprehensive R Archive Network (CRAN, https://cran.r-project.org).

### 2.5. Data Availability and Code

All datasets used in the present study are available open source at the links given in Table 1. The compiled and curated datasets are available in Github, together with the associated R Studio code (https://github.com/M-Di-Miceli/GP_practices accessed on 4 June 2025) for replication.

## 3. Results

As of December 2023, there were 6398 active GP practices in England, with a total of 62,454,328 registered patients. In Northern Ireland, 318 practices were in charge of 2,041,000 patients. In Scotland, 5,977,579 patients were registered in 901 practices. In Wales, 3,295,984 patients were registered in 348 practices.

**Figure 1:**
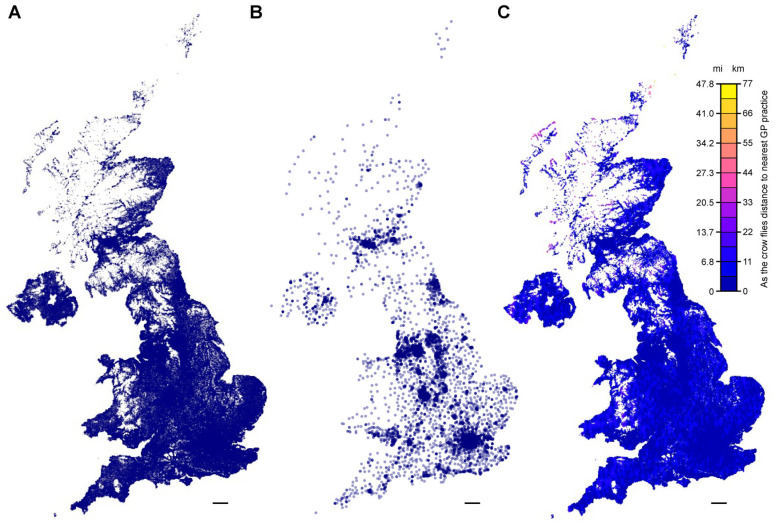
Geographical distances to GP practices in the UK. (**A**) Mapping of all valid postcodes. (**B**) Density mapping of all GP practices. (**C**) As-the-crow-flies distances between postcodes and nearest GP practice. Scale bars = 50 km.

In December 2023, 1,781,980 unique household postcodes were occupied by UK residents (Figure 1A). Whilst densely populated regions were observed in the main metropolitan areas, we also observed less dense areas, such as lakes, forest and mountainous regions. In parallel, active GP practices (with registered patients) were found throughout the UK (*n* = 7965), but with some great disparities in density (Figure 1B). Indeed, Wales, the Highlands and the North of England presented lower densities of GP practices. When computing as-the-crow-flies distances to the nearest GP practice, most UK postcodes (98.8%, *n* = 1,761,766) were within 10 km of a GP practice, with a minority (0.2%, *n* = 3675) at least 15 km away, a fraction (0.05%, *n* = 952) at least 20 km away and only an infinitesimal proportion (2.8 × 10^−6^, *n* = 5) further than 50 km away (Figure 1C). The median distance to the nearest GP in the UK was 0.97 km, with a mean of 1.75 km. Fourteen postcodes were computed as located more than 40 km from the nearest practice (postcodes starting in KW17 or ZE2). The postcode the furthest away from a GP practice (66.1 km from nearest practice) is located on an island in the south-west of the Shetland Islands. These postcodes can be seen on Figure 1C in warm colours (to the north-east of the map).

**Figure 2:**
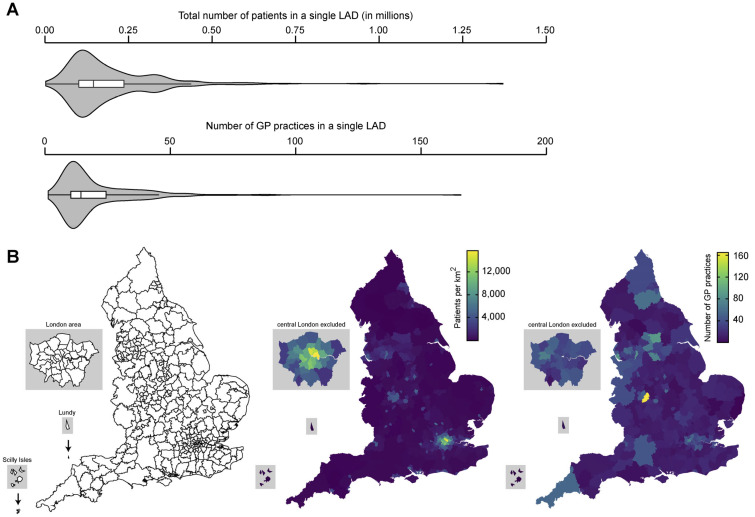
Population density and number of GP practices in England. (**A**) Total number of patients registered in LAD (*n* = 327, top), total number of GP practices in LAD (*n* = 327, bottom). (**B**) LADs in England (left), population density per km^2^ (middle) and number of GP practices (right). Central London was excluded from the analysis presented in (**B**). Grey rectangles present magnifications of the London metropolitan area, Lundy and Isles of Scilly.

In England, a total of 62,454,328 patients were registered in GP practices as of December 2023. The total number of GP practices across England were 6398, amongst 327 LADs. The maximum number of patients registered in a single LAD was in Birmingham, with 1,370,422 registered patients. The maximum number of GP practices in a single LAD was also in Birmingham (*n* = 165), likely accounting for such a high number of patients in this region of England. Finally, the maximum number of registered patients in a single GP practice was in Cumberland, with one practice responsible for the care of 37,836 patients. The area with the lowest number of registered patients was on the Isles of Scilly (*n* = 2426), with a single GP practice. The median total number of registered patients in an English LAD was 145,017 (mean = 191,096), and the median number of GP practices was 14 (mean = 19.57) (Figure 2A). To put these results in context of population density in different geographical areas, we mapped population density and number of GP practices in each LAD (Figure 2B). LAD in England presented varying population density, with a median of 639 persons per km^2^ (mean = 1744). The maximum density was found in Tower Hamlets, with 15,703 persons per km^2^ (appearing bright yellow and to the North of London in Figure 2B). The next top ten most densely populated LADs were Islington, Hackney, Lambeth, Kensington & Chelsea, Hammersmith & Fulham, Southwark, Newham, Camden, Wandsworth and Westminster. We found a weak correlation between population density and number of GP practices in the different LADs in England (r^2^ = 0.17, Supplementary Figure S1), likely due to primary care being organised in catchment areas [34] rather than pure geographical LAD boundaries.

**Figure 3:**
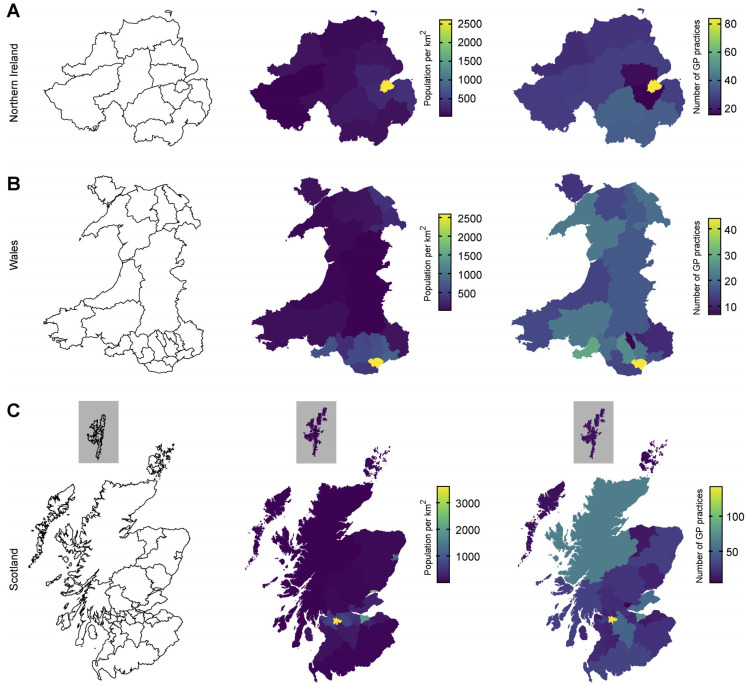
Population density and number of GP practices in Northern Ireland, Wales and Scotland. LADs in Northern Ireland (**A**), Wales (**B**) and Scotland (**C**) (left), with population density (in km^2^, middle) and number of GP practices (right). Grey rectangles are indicative of displacements from real geographical coordinates. Maps are not to scale with one another.

Similarly, in Northern Ireland, Wales and Scotland, large metropolitan areas had a high number of GP practices (Figure 3). The correlation between population density and total number of GP practices in each LAD was strong in Northern Ireland (r^2^ = 0.81), while Wales and Scotland only presented a medium correlation (r^2^ = 0.40 and r^2^ = 0.44, respectively). Data for the entire UK (327 LADs in England, 11 in Northern Ireland, 32 in Scotland and 22 in Wales) indicated (Supplementary Figure S1) only a weak overall correlation (r^2^ = 0.13, *p* = 8.2 × 10^−14^) between population density and number of GP practices.

## 4. Discussion

The present study highlights that almost all residents in the UK are within 10 km of a GP practice (proximity based on centroid-to-centroid distance). In addition, computed data on density of patients across LADs indicate a weak to strong correlation with number of GP practices.

In an observational study conducted in England in 2007, distance was directly linked to mortality. Indeed, every 10 km of an as-the-crow-flies distance of an ambulance journey was correlated to a 1% increase in mortality in patients with life-threatening emergencies, with respiratory events showing the greatest association [35]. Another study in England and Wales found that older patients tend to have longer distances to travel between home and hospital, but distance was not reported as a significant risk for 30-day mortality after urgent laparotomy procedures [36]. In Denmark, distance to GP practice was not associated with tumour stage, but distance to diagnostic facilities or hospital was [37]. However, the same study also highlighted a negative correlation between distance to hospital and disease stage at diagnostic for cancers with poor survival rates [37]. In contrast, another study in the North of England concluded that distance to GP practice was associated with later stage diagnoses for breast or colorectal cancers, whilst distance to hospital was not [38]. In countries from the Global North, an overall association between distance to primary or secondary healthcare facilities and health outcomes was observed [39], thus highlighting the need to improve distance to GP practices for better public health. It is also important to note that GP practices are often (73.5%) involved in the initial diagnostic of cancer [40]. A recent prospective study from Denmark identified distance to GP practice as a significant factor for not having received a face-to-face appointment with a GP before acute myocardial infarction [41].

In the UK, almost 85% of residents (84.8%) are within walking distance to the nearest GP practice [18], showing good geographical distance for most patients. However, this is not the case for rural areas, where only 19.2% of the population is within a 20 min walk of a GP practice [18]. Nonetheless, some patients might not be able to walk to the nearest GP practice, and some patients might register with a practice that is not the closest to their residence. In fact, a survey conducted in 2025 reported that 63% of patients are either walking or cycling to the GP practice, with 38% of patients driving [42], although no specifics were given for the methodology or population surveyed. In East Anglia, 67% of the population lived less than a 5 min drive to the nearest GP practice, with 10% living more than 10 min away, and 13% of patients not being able to use the bus easily [43], further highlighting geographical disparities amongst the population, at least in 2002. In Northern Ireland, only 19% of all super output areas have population residing within 1.5 km of a GP practice, with 24% of all super output areas having no patients within 1.5 km of a GP practice [16].

The current study reported increased resolution to previous reports on straight-line distance to nearest GP practice. Our analyses computed straight-line geographical distances to nearest GP practice for every single postcode in the UK. In addition, we also examined the relationship between population density and number of GP practices throughout the UK, thus providing quantifiable data. To conclude, we suggest a few adjustments to improve geographical distance to the nearest GP practice in specific areas, such as:

1. in the most southern and western isles from the Shetland Islands.
2. for postcodes starting in KW17 and ZE2.

### Limitations of This Study

A limitation identified in the present study is the use of valid postcodes from December 2023. Since December 2023, new postcodes have been created by Royal Mail, while some have been terminated. Therefore, our current analyses are slightly behind. However, one can assume that most new residences are created within geographical areas in proximity to local amenities, including healthcare facilities.

Another potential limitation is that we were unable to compute real distances to nearest GP practice. Indeed, our study used as-the-crow-flies distances, which are not necessarily a good proxy to actual road distances nor travel time. For example, mountains, lakes, rivers, forests and natural obstacles can greatly increase the distance needed to be travelled when compared to flying distance, as evidenced previously [44,45,46,47]. Furthermore, travel time is not always correlated to distance, as volume of traffic can greatly influence travel time. In fact, an alternative approach to account for such variations between road distance and actual travel time is to use floating catchment area techniques. These consist of accounting for road patterns, road types (earth, tarmac), speed limitations and mode of transportation (walking, driving, public transport, etc…). These variables are considered the minimum needed to effectively calculate travel time in addition to distance to reach healthcare facilities. Such a model is termed “2FCA”, for 2-step floating catchment area, consisting of a first step of quantifying number of residents within a certain threshold distance, followed by calculating availability of healthcare facilities in that threshold distance [48,49]. These calculations are then combined into an accessibility score. The 3FCA (3-step floating catchment area) model was then created based on the 2FCA, with the introduction of a new variable accounting for residents’ choice in healthcare providers [50]. This model introduced a distance decay parameter, reflecting that residents are more likely to select a healthcare provider closer to their residence. Another method which is often used is the “enhanced” version of the 2/3FCA models, in which a dynamic iteration of population recruitment is performed until a determined threshold is reached based upon available healthcare facilities in the vicinity [51]. Finally, some authors also decide to perform hierarchical modelling using these different models, consisting of flexible healthcare demands in the catchment population (primary, secondary, tertiary facilities) [52]. Some examples of these techniques include measuring access (i.e., more than just distance) to primary care [53] or healthcare facilities [52,54]. Whilst these techniques offer more precise accessibility assessments by taking into account several variables, these require complex computations which heavily depend on data availability (road networks, public transport maps, traffic density by hour, oversimplification of healthcare demands, etc…).

Finally, we acknowledge that catchment areas for primary care are not governed by LAD geographical boundaries [34], and some GP practices may serve patients who live in a different LAD than the one in which the practice is located.

## 5. Conclusions

In the UK, the relationship between population density and GP practice numbers varied across LADs, ranging from weak to strong correlations depending on the region examined. This variability indicates that population density alone does not fully explain the distribution of primary care infrastructure and highlights the influence of additional demographic factors. Furthermore, as-the-crow flies distance to nearest GP practice was found to be within 10 km for 98.8% of all UK residents. Collectively, these findings provide an updated national overview of spatial distance to GP services and establish a baseline for future assessments of primary care accessibility and healthcare equity across the UK.

While geographical proximity is an important component of healthcare access, distance alone does not capture the full complexity of primary care accessibility. Future studies should incorporate measures of GP workforce capacity, including the number of full-time equivalent clinicians, appointment availability and patient-to-practitioner ratios (including nurses and additional support staff), to better assess the relationship between service provision and population need. In addition, accessibility analyses would benefit from the inclusion of transport-related factors. Road-network travel times, public transport availability and connectivity in rural and underserved areas may substantially influence practical access to primary care services, particularly among older adults and socioeconomically disadvantaged populations. Integrating these variables into spatial accessibility models would provide a more nuanced understanding of barriers to primary care. Further research could also examine temporal trends in GP practice distribution, closures, mergers and population growth, in addition to investigating health outcome metrics. Such work would help identify areas where service provision may be insufficient and support evidence-based planning aimed at reducing inequalities in access to primary healthcare across the UK.

## Figures and Tables

**Table 1:** Publicly available open-access datasets used in the present study. CSO: Central Statistics Office; DSC: Data Stirling Council, OfNS: Office for National Statistics, NHSD: NHS Digital, PHS: Public Health Scotland, OGP: Open Geography Portal, HSCS: Health and Social Care Northern Ireland, DMW: DataMapWales, WG: Welsh Government, LAD: local area districts.

Provider	Link	Description
NHSD	https://digital.nhs.uk/data-and-information/data-tools-and-services/data-services/general-practice-data-hub (accessed on 4 June 2025).	Data on GP practices in England
PHS	https://publichealthscotland.scot/healthcare-system/primary-care/general-practice/general-practice-data/general-practice-list-size-and-demographics-information/ (accessed on 4 June 2025).	Data on GP practices in Scotland
DMW	https://datamap.gov.wales/layergroups/geonode:gp_sites_ogl (accessed on 4 June 2025).	Data on GP practices in Wales
HSC	https://bso.hscni.net/directorates/operations/family-practitioner-services/medical-services/contractor-information/northern-ireland-gp-practice-lists-for-professional-use/ (accessed on 4 June 2025).	Data on GP practices in Northern Ireland
OGP	https://geoportal.statistics.gov.uk/datasets/80592949bebd4390b2cbe29159a75ef4/about (accessed on 4 June 2025).	Postcodes in the UK
OfNS	https://geoportal.statistics.gov.uk/datasets/ba0873184e6349bebb63b5da6dd050b5/about (accessed on 4 June 2025).	Administrative area measurements
NHSD	https://digital.nhs.uk/data-and-information/publications/statistical/patients-registered-at-a-gp-practice/may-2023 (accessed on 4 June 2025).	Additional data on GP practices in England
HSC	https://datavis.nisra.gov.uk/bso/general-medical-statistics-2022-2023.html (accessed on 4 June 2025).	Additional data on GP practices in Northern Ireland
WG	https://www.gov.wales/general-practice-workforce-31-december-2023 https://statswales.gov.wales/Catalogue/Health-and-Social-Care/General-Medical-Services/General-practice-population/patients-registered-at-a-gp-practice (accessed on 4 June 2025).	Additional data on GP practices in Wales
PHS	https://publichealthscotland.scot/publications/general-practice-demographics-data-visualisation/general-practice-demographics-data-visualisation-up-to-31-december-2023/ (accessed on 4 June 2025).	Additional data on GP practices in Scotland
OGP	https://geoportal.statistics.gov.uk/datasets/local-authority-districts-december-2019-boundaries-uk-bgc/explore (accessed on 4 June 2025).	LAD *shapefile*
OGP	https://geoportal.statistics.gov.uk/datasets/a2f8c9c5778a452bbf640d98c166657c/about (accessed on 4 June 2025).	Postcode coordinates
OfNS	https://www.ons.gov.uk/datasets/TS006/editions/2021/versions/4 (accessed on 4 June 2025).	Population density by LAD in England
CSO	https://data.cso.ie/table/CPNI06 (accessed on 4 June 2025).	Population density by LAD in Northern Ireland
HSC	https://bso.hscni.net/directorates/operations/family-practitioner-services/directorates-operations-family-practitioner-services-information-unit/1776-2/ (accessed on 4 June 2025).	Additional data on GP practices in Northern Ireland
OfNS	https://www.nomisweb.co.uk/query/select/getdatasetbytheme.asp?opt=3&theme=&subgrp= (accessed on 4 June 2025).	Population density by LAD in Wales
DSC	https://data-stirling-council.hub.arcgis.com/datasets/stirling-council::population-density-national-records-of-scotland-2023-mid-year-estimates-open-data/explore (accessed on 4 June 2025).	Population density by LAD in Scotland

## Data Availability

All compiled datasets and code necessary to replicate the current results, including figures, have been placed in an open-access repository at Github. The link is provided in the methods section and is reproduced here: https://github.com/M-Di-Miceli/GP_practices accessed on 4 June 2025. Readers wanting to replicate the current study are strongly advised to start with the Readme text (https://github.com/M-Di-Miceli/GP_practices/blob/main/README.md accessed on 4 June 2025), which contains some instructions. The compiled datasets can be accessed using this link: https://github.com/M-Di-Miceli/GP_practices/tree/Datasets accessed on 4 June 2025 while the code necessary to reproduce the results presented in the current study can be accessed using this link: https://github.com/M-Di-Miceli/GP_practices/tree/Code accessed on 4 June 2025.

## Supplementary Materials

The following supporting information can be downloaded at: https://www.mdpi.com/article/10.3390/epidemiologia7030085/s1, Figure S1: Relationship between population density (people/km^2^) and number of GP practices. Each point corresponds to a local area district, colour-coded for the different countries in the United Kingdom. Note the logarithmic scales on both axes. E: England, NI: Northern Ireland, S: Scotland and W: Wales.
