# Supplementary material for: Geospatial Visualisation of Distance to General Practitioner Facilities with Population Density Patterns in the United Kingdom
Source: Epidemiologia (Basel). 2026 Jun 17;7(3):85. doi: 10.3390/epidemiologia7030085 (PMC13298430; doi:10.3390/epidemiologia7030085)
Supplement: Supplementary file 1 [file epidemiologia-07-00085-s001.zip › epidemiologia-4223025-supplementary.pdf]

epidemiologia

MDPI

*Supplementary Documents*

# Supplementary documents - Geospatial Visualisation of Distance to General Practitioner facilities with Population Density Patterns in the United Kingdom.

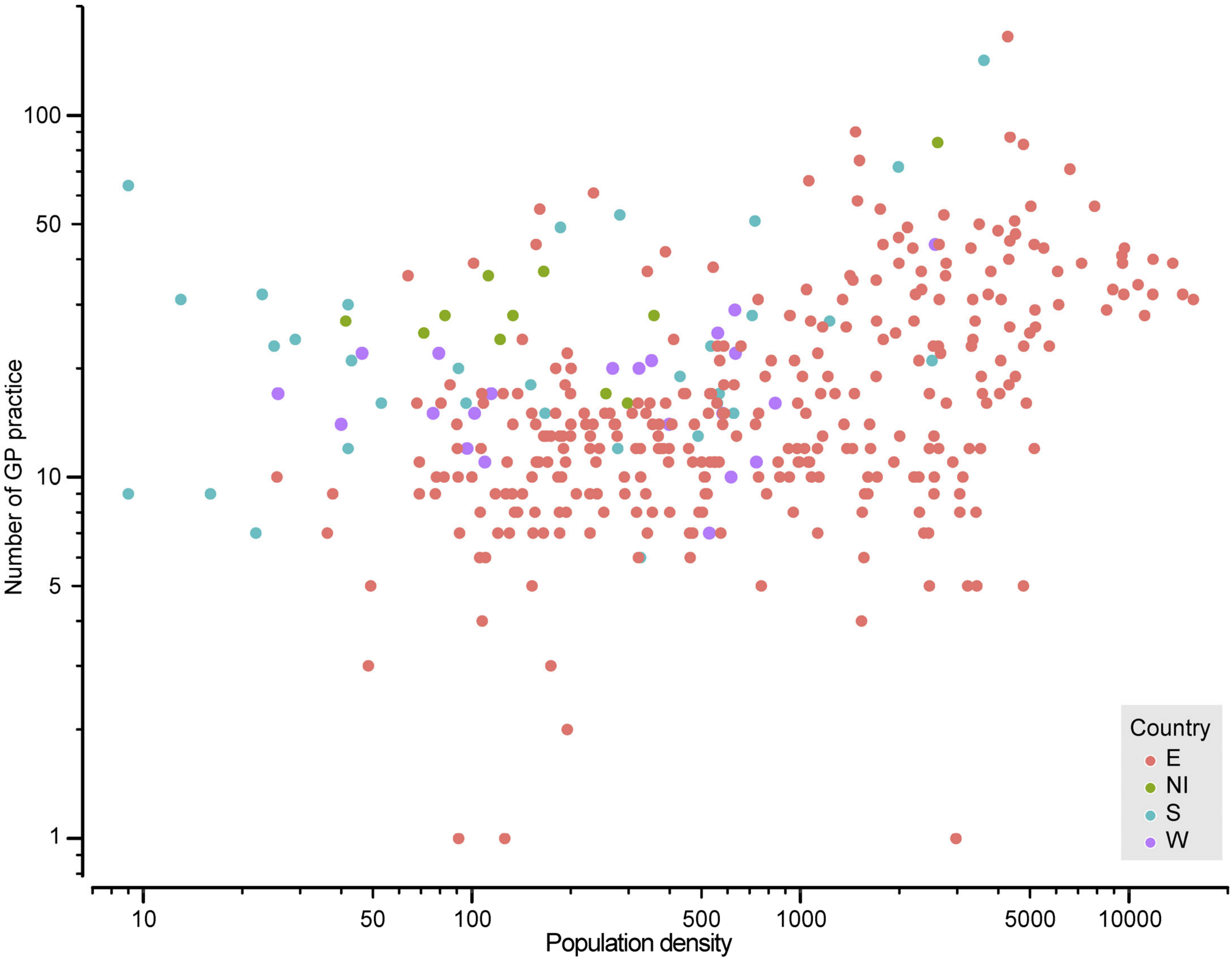


**Supplementary Figure S1.** Relationship between population density (people/km$^2$) and number of GP practices. Each point corresponds to a local area district, colour-coded for the different countries in the United Kingdom. Note the logarithmic scales on both axes. E: England, NI: Northern Ireland, S: Scotland and W: Wales.

**Disclaimer/Publisher's Note:** The statements, opinions and data contained in all publications are solely those of the individual author(s) and contributor(s) and not of MDPI and/or the editor(s). MDPI and/or the editor(s) disclaim responsibility for any injury to people or property resulting from any ideas, methods, instructions or products referred to in the content.
